# Clinical and Microbiological Characteristics of Mycobacterium kansasii Pulmonary Infections in China

**DOI:** 10.1128/spectrum.01475-21

**Published:** 2022-01-12

**Authors:** Yinjuan Guo, Yanhua Cao, Haican Liu, Jinghui Yang, Weiping Wang, Bingjie Wang, Meilan Li, Fangyou Yu

**Affiliations:** a Department of Clinical Laboratory, Shanghai Pulmonary Hospital, School of Medicine, Tongji University, Shanghai, China; b Department of Respiratory Intensive Care Unit, Shanghai Pulmonary Hospital, School of Medicine, Tongji University, Shanghai, China; c State Key Laboratory of Infectious Disease Prevention and Control, Chinese Center for Disease Control and Prevention, Beijing, China; Johns Hopkins University School of Medicine

**Keywords:** *Mycobacterium kansasii*, drug sensitivity, genotype

## Abstract

Mycobacterium kansasii, an important opportunistic pathogen of humans, causes serious pulmonary disease. Sixty M. kansasii isolates were collected for investigating the clinical characteristics of patients with M. kansasii infections as well as drug susceptibility and genotypes of M. kansasii. More than 90% of the patients infected with M. kansasii were from eastern China. According to the internal transcribed spacers (ITS), *rpoB*, *hsp65*, and *tuf*, all M. kansasii isolates were classified as molecular type I, irrespective of the disease manifestation. Sixty M. kansasii isolates from China were diverse and separated into four branches. Pairwise average nucleotide identity (ANI) values for M. kansasii isolates affiliated with different genotypes were more than 85%. The earliest isolate was isolated from Jiangsu in 1983. Of the isolates, 78.3% (47/60) were isolated since 1999. All isolates were sensitive to rifabutin. All but one isolate was sensitive to clarithromycin. Sensitivity rates to rifampin, amikacin, moxifloxacin, and linezolid were 80.0%, 90.0%, 88.3%, and 91.7%, respectively. A high rate of resistance was noted for ciprofloxacin (44 isolates, 73.3%) and ethambutol (46 isolates, 76.7%). Compared with M. tuberculosis H37Rv, 12 mutations of *embCA* were observed in all M. kansasii isolates. All these 60 M. kansasii isolates shared identical sequences of *rpoB*, *inhA*, *katG*, *rrl*, *rrs*, *rpsL*, *gyrA*, and *gyrB*. In conclusion, M. kansasii isolates are exhibiting greater genetic diversity globally. The resistance mechanism of M. kansasii is not necessarily related to gene mutation.

**IMPORTANCE** M. kansasii type I is the main genotype spreading worldwide. The molecular history of the global spread of type I isolates remains largely unclear. We conducted a detailed analysis of genomic evolution of global M. kansasii isolates. Our results suggest that M. kansasii isolates exhibit greater genetic diversity globally.

## INTRODUCTION

Mycobacterium kansasii, a slow-growing nontuberculous mycobacterium (NTM), is one of the most pathogenic and common NTMs isolated from humans (1). To date, seven genotypes (I–VII) have been identified, along with two intermediate (I/II) and atypical (IIb) types (2, 3). Of these, I and II are the most prevalent types that have been associated with NTM-pulmonary disease, while the others have usually been linked to environmental sources (3). There are three major methodologies used for the identification of M. kansasii subtypes, including sequence analysis of either *rpoB* or *hsp65* genes, 16S-23S rDNA internal transcribed spacers (ITS), and *tuf* typing (4).

The prevalence of M. kansasii diseases has varied widely by region and over time. In Slovakia, the United Kingdom, and Poland, a high prevalence of M. kansasii infections of all NTM isolates had been consistently reported (36%, 11%, and 35%, respectively) (5). Likewise, Brazil, Japan, and South Africa have reported high M. kansasii rates of infection of all NTM isolates (6). However, the prevalence of M. kansasii infections of all NTM isolates was low in Europe, with a mean isolation rate of 5% (5). M. kansasii infection is also more common in human immunodeficiency virus (HIV)-infected individuals. The annual incidence of M. kansasii infection in people living with HIV may be as high as 5.32%, compared with 0.005% in a survey of 44 U.S. states prior to the HIV epidemic (7–10). M. kansasii is also the second most frequent NTM found in HIV-infected patients.

M. kansasii pulmonary infection often presents with a clinical syndrome indistinguishable from that of M. tuberculosis; the most common presenting symptoms are cough, chest pain, dyspnea, and nonmassive hemoptysis (11, 12). In addition, common sites of M. kansasii extrapulmonary disease include the lymph nodes, skin, and musculoskeletal and genitourinary systems (1).

American Thoracic Society/Infectious Disease Society of America (ATS/IDSA) guidelines recommend daily therapy with isoniazid, rifampin, and ethambutol when the disease is fully sensitive to these drugs (11). For use only in the rare event of resistance to rifampin, alternative medications that are active against M. kansasii include streptomycin, clarithromycin, amikacin, ethionamide, sulfamethoxazole, rifabutin, linezolid, and fluoroquinolones. To date, *in vitro* drug susceptibility tests are standardized for only a few species of NTM. The resistance breakpoints of isoniazid and streptomycin to M. kansasii are not listed in the Clinical and Laboratory Standards Institute (CLSI) M24-A2 (13), and the genetic determinants of drug resistance in M. kansasii are virtually absent. In Mycobacterium tuberculosis, the genetic determinations of drug resistance are well characterized. The majority of drug resistance in clinical Mycobacterium tuberculosis strains is attributed to chromosomal mutations (14). Resistance related mutations could also exert certain fitness cost to the drug-resistant Mycobacterium tuberculosis strains, and growth fitness could be restored by the presence of compensatory mutations (14, 15).

The purpose of this study was to investigate the molecular identification and determination subtypes, clinical characteristics, geographic distribution, and drug susceptibility of clinical isolates of M. kansasii in China.

## RESULTS

### Demographic data and clinical characteristics.

**FIG 1:**
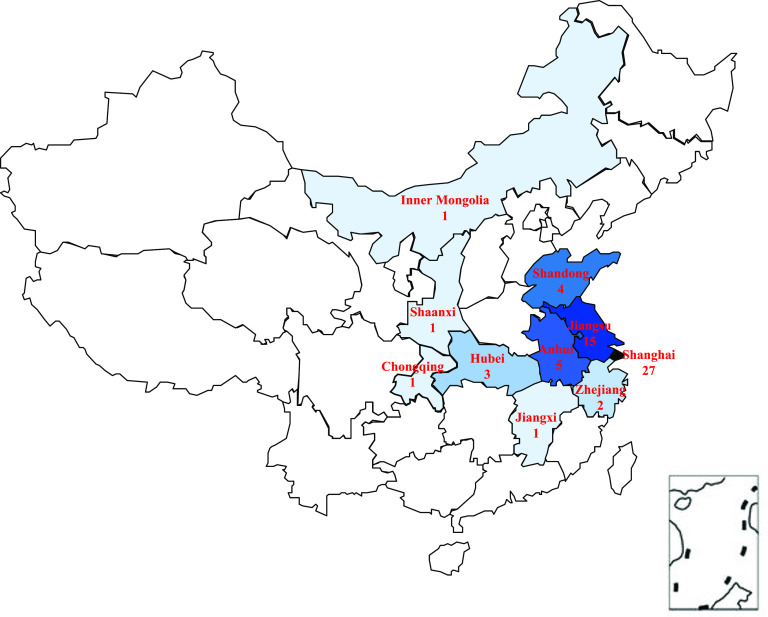
Map of China showing the distribution of 60 M. kansasii isolates included in this study (the numbers indicate the number of isolates in each region).

A total of 60 M. kansasii strains were isolated from patients. Fig. 1 shows the geographical distribution of these isolates. More than 90% of the patients were from eastern China. Characteristics of the study group are shown in Table 1. The mean age of patients was 43.7 ± 24.6 years, ranging from 17 to 85 years. Patients younger than 50 years accounted for 75% of the study population. Of the 60 patients, 44 (73.3%) were male. None of the medical records mentioned HIV/AIDS-positive status. The most common presenting symptom was cough, reported in 75.0% of patients. It was followed by sputum (61.7%) and hemoptysis (28.3%).

**TABLE 1:** Clinical characteristics of 60 patients with M. kansasii infection^*a*^

Characteristics	Data (*n*, %)
Age, yrs (mean ± SD)	43.7 ± 24.6
Sex	
Male	44 (73.3)
Female	16 (26.7)
Associated lung disease	
Previous tuberculosis	13 (21.7)
COPD	9 (15.0)
Bronchiectasis	5 (8.3)
Symptoms	
Chest pain	9 (15.0)
Cough	45 (75.0)
Sputum	37 (61.7)
Hemoptysis	17 (28.3)
Febrile sense	15 (25.0)
Radiologic findings	
Nodule	49 (81.7)
Cavity	40 (66.7)
Opacities	52 (86.7)
Infiltrations	38 (63.3)
Pleural thickening	21 (35.0)

a SD, standard deviation; COPD, chronic obstructive pulmonary disease.

Associated lung diseases included COPD (9 patients, 15.0%), previous tuberculosis (13 patients, 21.7%), and bronchiectasis (5 patients, 8.3%). Radiological analysis revealed that pulmonary opacities and infiltrations were the dominant patterns in patients with M. kansasii infection (86.7% with opacities form and 63.3% with infiltrations form). Cavities and nodules were observed in approximately two-thirds of patients.

### Genotypes.

**FIG 2:**
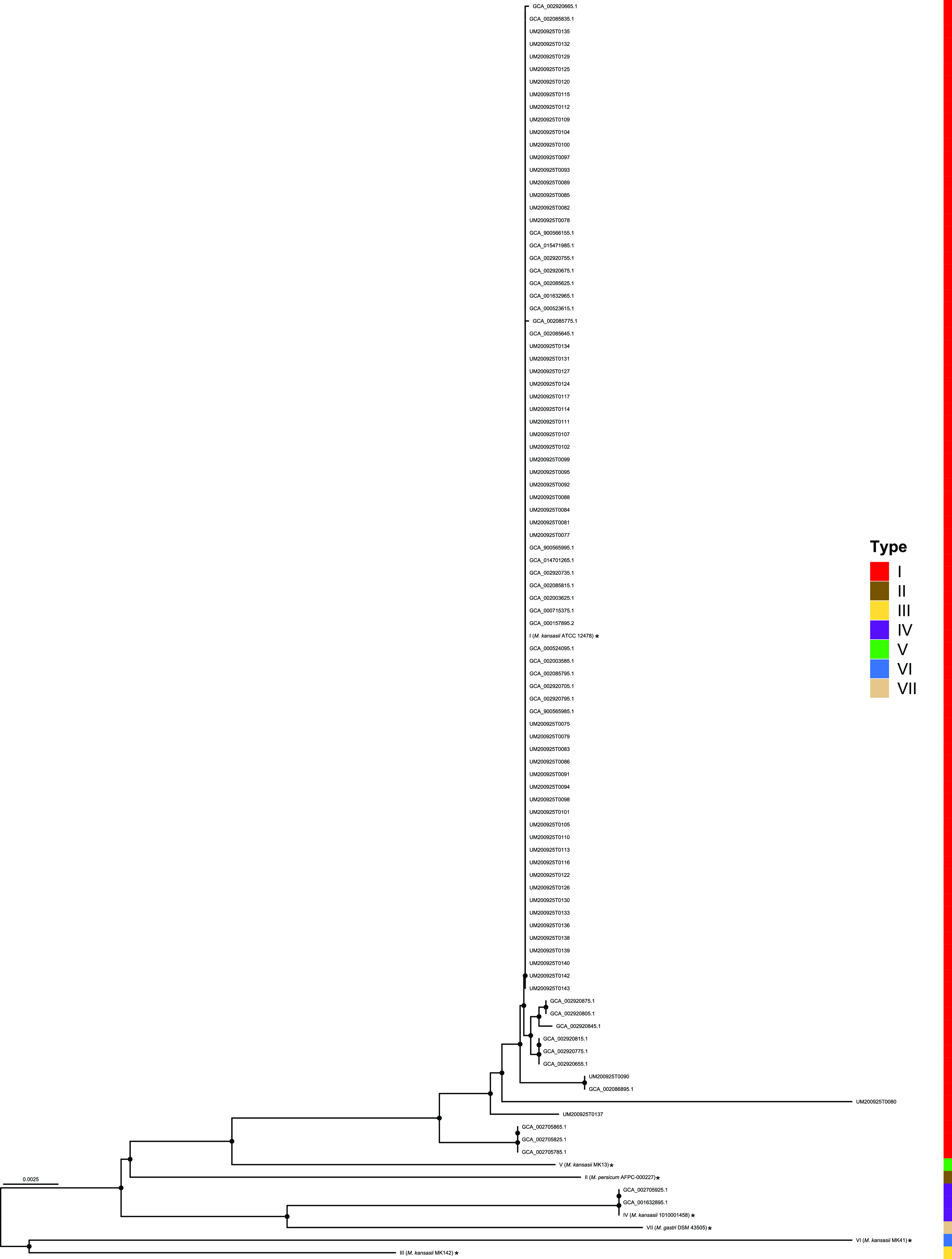
Phylogenetic tree based on *rpoB*, *hsp65*, internal transcribed spacers (ITS), and *tuf* genes sequences constructed using the neighbor-joining method. The bootstrap values were calculated from 1,000 replications.

All 60 isolates tested were classified as M. kansasii type I based on *rpoB* and *hsp65* gene sequences (Fig. S1A and B in the supplemental material). Fifty-nine isolates exhibited 98.3% identity at both loci when compared with the M. kansasii genotype I reference strain (ATCC12478) based on ITS and *tuf* gene sequences (Fig. S1C and D). The polygenetic tree that was constructed based on the *tuf* and ITS grouped the M. kansasii subtypes incorrectly in some cases. M. kansasii isolate UM200925T0090 from this study was identified as M. kansasii genotype II based on the ITS sequence. M. kansasii isolate UM200925T0137 from this study was identified as M. kansasii genotype II based on the *tuf* gene. M. kansasii isolate UM200925T0080 has not been typed based on the *tuf* gene. A separate tree was created using the concatenated *rpoB*, *hsp65*, ITS, and *tuf* genes (Fig. 2). In addition, M. kansasii isolate GCA_002086895.1 isolated from the Netherlands was genotype II based on the ITS sequence, but genotype I according to the other method. Three isolates isolated from South Korea (GCA_002705825.1, GCA_002705865.1, and GCA_002705785.1) were genotype II based on *hsp65* sequence, while classified as type I based on the ITS, *rpoB*, and *tuf*.

**FIG 3:**
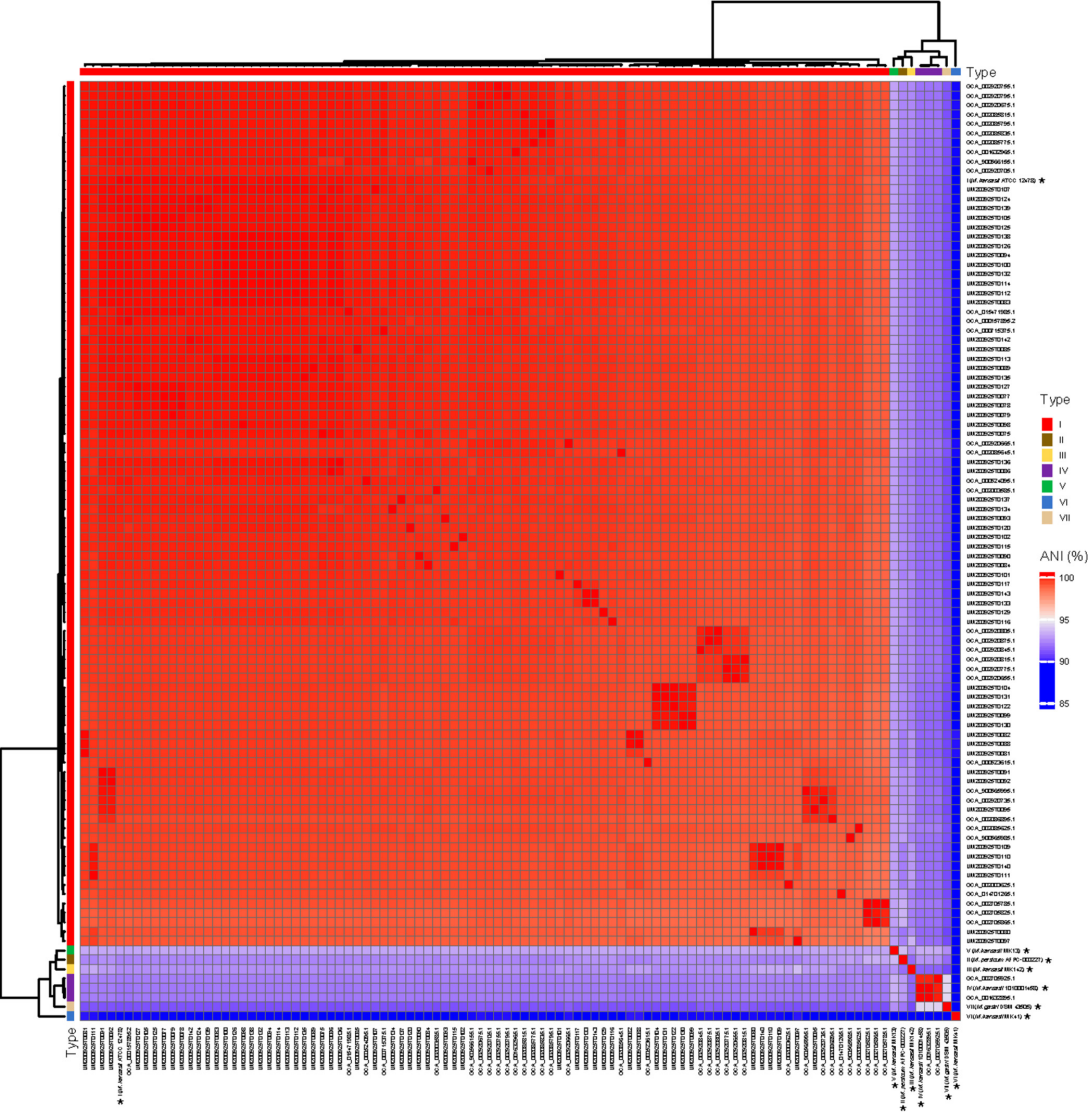
Pairwise comparisons of ANIs of M. kansasii subtypes I-VII.

The ANI values for isolates of the same M. kansasii genotype were close to 100% (Fig. 3). Pairwise ANI values for M. kansasii isolates affiliated with different genotypes were more than 85%.

### Phylo-geographical context and comparative genomics of M. kansasii.

**FIG 4:**
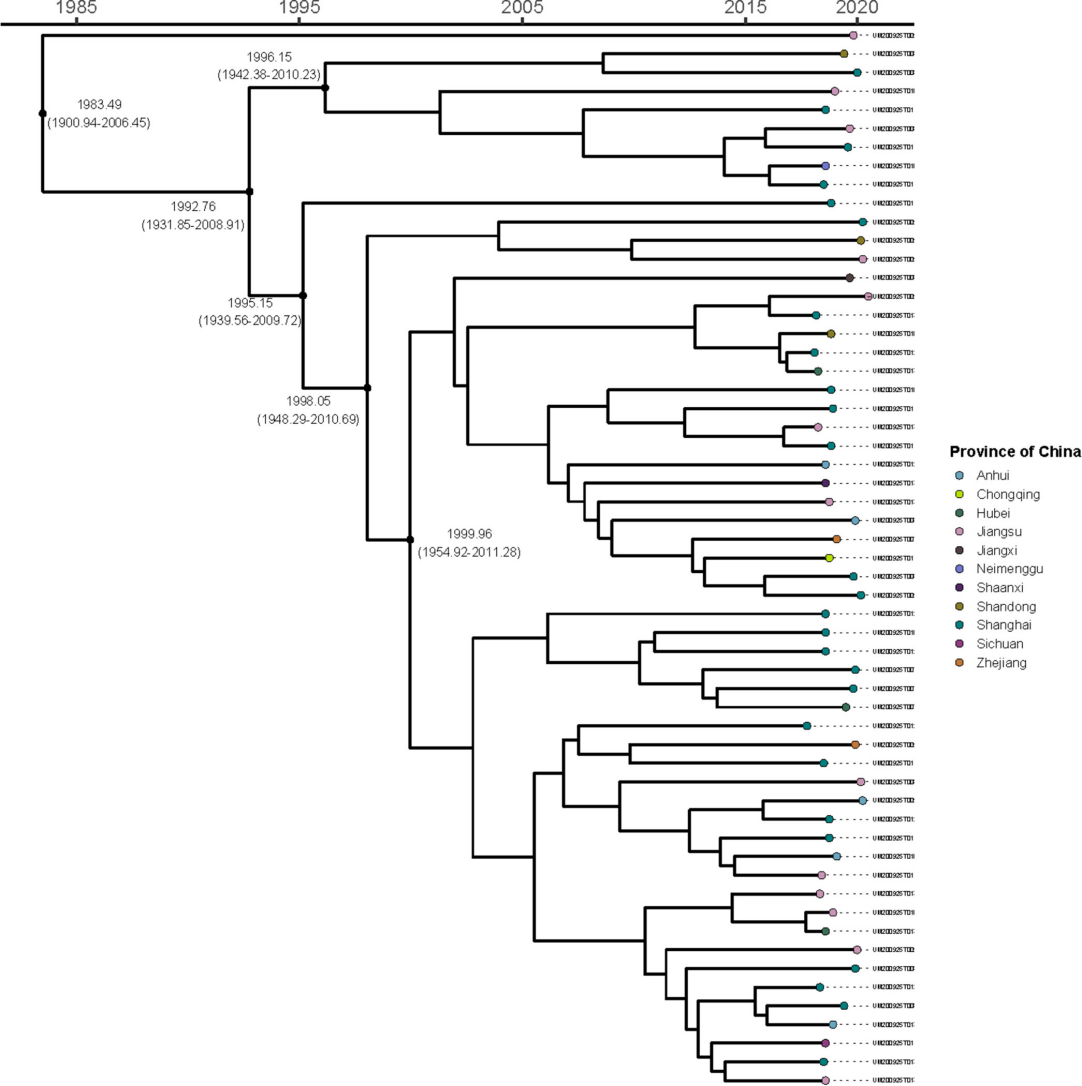
The output from Gubbins was used as the input for BactDating v1.0 to perform phylogenetic dating based on a Bayesian approach. The Markov chain Monte Carlo chain lengths were run for 100 million cycles to convergence; the effective sample size of the inferred parameters α, μ, and σ was >200.

To analyze the evolution of the M. kansasii from China, we determined the genome sequences of 60 *M. kansaii* isolates. The genomes of 36 M. kansasii isolates (supplemental file 2) previously reported by NCBI (https://www.ncbi.nlm.nih.gov/genome/browse/#!/prokaryotes/1793/) were included for analysis to gain a better understanding of the M. kansasii genome globally. Phylogenetic analysis and divergence time estimation among 60 M. kansasii isolates are shown in Fig. 4. The earliest isolate was from Jiangsu in 1983. Of the isolates, 78.3% (47/60) were isolated since 1999.

**FIG 5:**
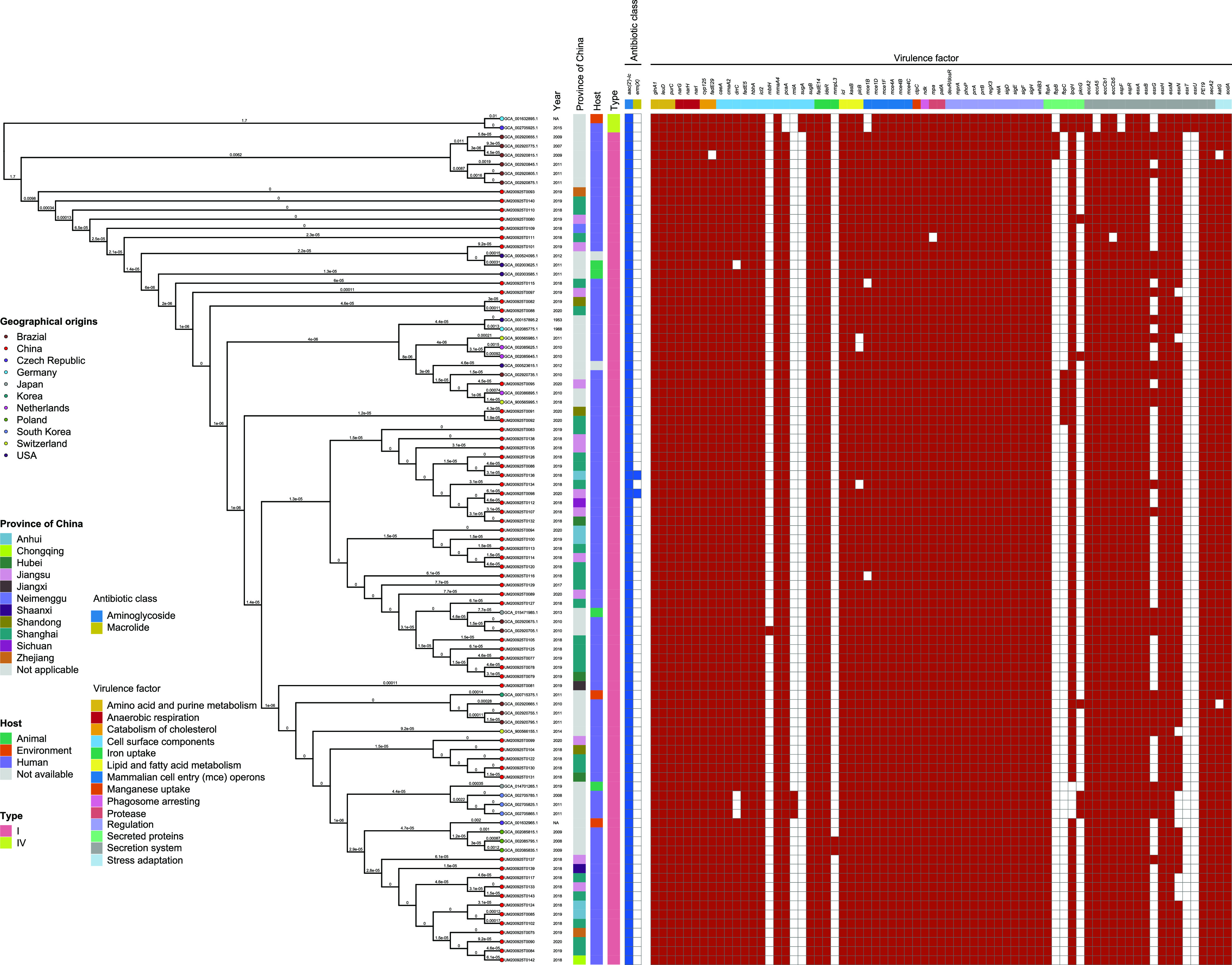
Phylogenetic analysis of 96 M. kansasii isolates. A phylogenetic tree was constructed using the SNPs outside of the recombination regions with RAxML using a GTR model and gamma correction. Colors in columns illustrate region of origin, mainland China provinces, host, type, antibiotic class, and virulence factor.

M. kansasii isolates exhibit greater genetic diversity globally (Fig. 5). Our analysis divided the 96 genomes into three main groups when compared to the genome of the ATCC isolate, one presenting less than 200 single nucleotide polymorphisms (SNPs; *n* = 88), a second group with either more than 1,500 SNPs and less than 2,000 SNPs (*n* = 6), and the third group with more than 10,000 SNPs (*n* = 2). SNPs are shown in supplemental file 3. The 60 M. kansasii isolates isolated from this study presented homogeneously distributed SNPs over the entire genome. Although genomic comparisons of 8 isolate from Brazil and Germany revealed greater heterogeneity, 60 isolates had fewer than 20 SNPs compared to the reference ATCC12478 isolate. A threshold of 4 SNPs will be best suited to fit the clustering from this study. SNP diversity among phylogenetically linked samples from this study ranged from 0 to 20 SNPs. Our analysis divided the 60 genomes into 4 main clusters, one presenting less than 4 SNPs, a second group with less than 5 SNPs and more than 10 SNPs, a third group with more than 11 SNPs and less than 15 SNPs, and a fourth group with more than 15 SNPs.

Sixty M. kansasii isolates from China were diverse and separated into four branches. Among the 60 M. kansasii isolates, 45 isolates (75.0%) belonged to the same branch and were homologous to 45.7% (16/35) of M. kansasii isolates in the NCBI database. Twelve M. kansasii isolates from Brazil belonged to four branches, which was the same as in a previous study (16). M. kansasii isolates from Poland (3 isolates), South Korea (3 isolates), and the Czech Republic (1 isolate) were more closely related.

### Virulence factor-encoding gene in M. kansasii genomes.

All virulence genes that met the filter threshold were listed (the filter threshold refers to the reference virulence gene, and homologous genes should have a coverage of ≥85% and a similarity of ≥85%). Virulence factor-encoding genes are listed in Fig. 5. Compared with M. kansasii genotype I, *pcaA*, *mce1B*, *fbpB*, *eccA5*, and *espF* genes were missing in M. kansasii genotype IV isolates. The *sugA*, *fbpC*, *pknG*, *esxU*, and *esxT* genes were absent in M. kansasii genotype I isolates. The *esxG* gene was absent in 56 M. kansasii isolates isolated from this study.

### Drug susceptibility pattern.

**FIG 6:**
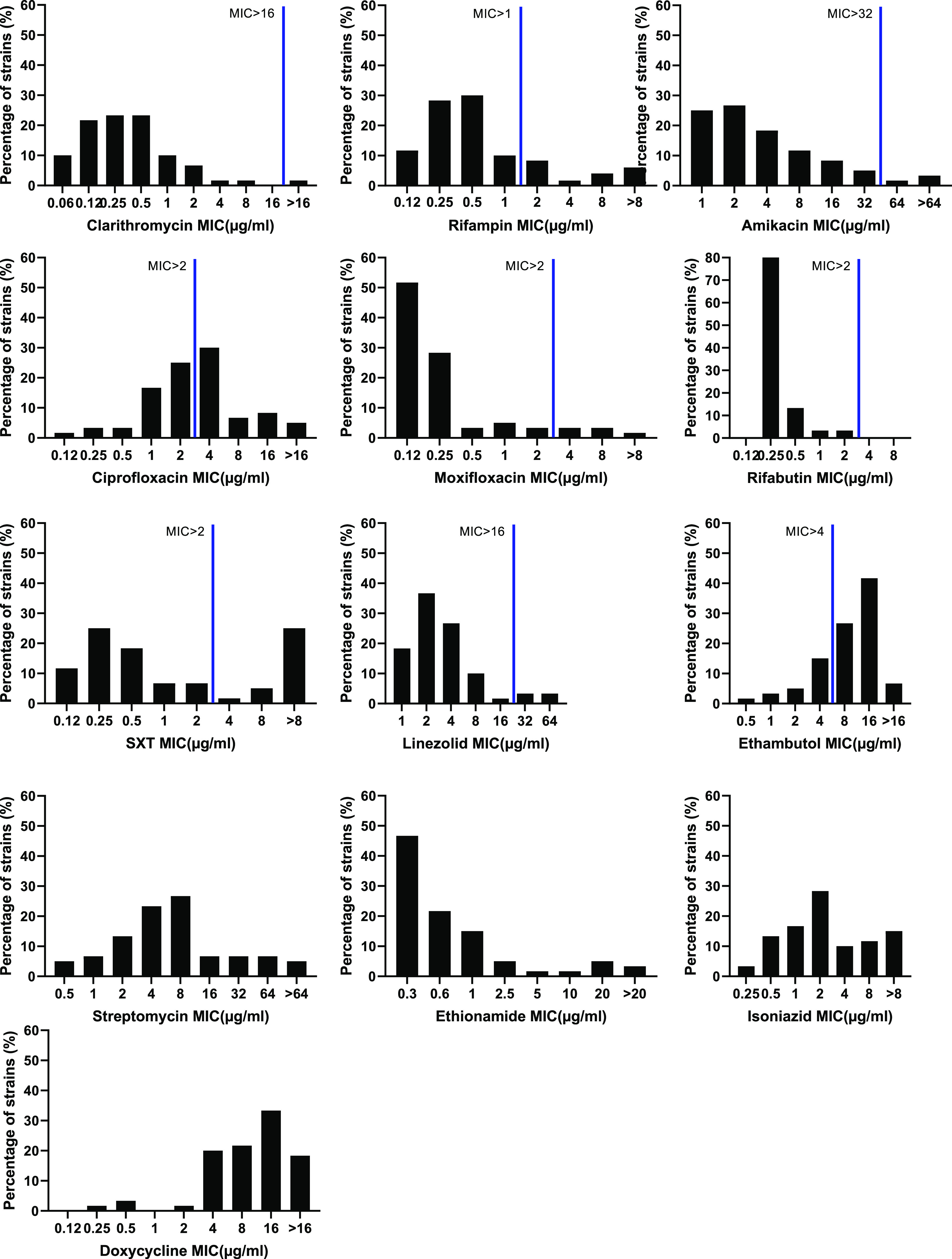
MIC distributions of 60 M. kansasii clinical isolates. Solid lines: tentative species-specific epidemiological cut-off (ECOFF) values (according to CLSI). No solid lines: no breakpoints were applicable.

MIC ranges, MICs, MIC_50_, and MIC_90_ (MIC required to inhibit the growth of 50% and 90% of the strains, respectively) are summarized in Table 2. All isolates were sensitive to rifabutin (RFB), and all but one isolate was sensitive to clarithromycin (CLR). Sensitivity rates of 60 isolates tested to rifampin (RIF), amikacin (AMK), moxifloxacin (MXF), and linezolid (LNZ) were 80.0%, 90.0%, 88.3%, and 91.7%, respectively. There were high resistance rates to ciprofloxacin (CIP; 73.3%, 44/60) and ethambutol (EMB; 76.7%,46/60). Four of the 13 antimicrobials tested, S, INH ETH, and DOX, have no cutoff point established by CLSI, so it was not possible to classify the samples as susceptible or resistant. MIC distributions of M. kansasii are shown in Fig. 6. The highest detected MIC for INH and S was >8 μg/mL and was found in 9 (15.0%) and 15 (25.0%) M. kansasii isolates, respectively. The MIC_90_ for S, INH, and DOX varied greatly (32 μg/mL, 8 μg/mL, and 16 μg/mL, respectively). However, ETH was highly active against M. kansasii, with MIC_50_ and MIC_90_ values of 0.6 μg/mL and 2.5 μg/mL, respectively.

**TABLE 2:** MIC ranges, MIC_50_, MIC_90_, and drug resistance in 60 clinical M. kansasii isolates from China

Antimicrobial agents	Tested concn ranges (μg/mL)	Breakpoints (μg/ml)	Resistance (%, *n*)	Susceptibility (%, *n*)	MIC_50_ (μg/mL)	MIC_90_ (μg/mL)
Primary agents						
Clarithromycin	0.06–16	>16	1.67(1)	98.3(59)	0.25	2
Rifampin	0.12–8	>1	20.0(12)	80.0(48)	0.5	4
Secondary agents						
Amikacin	1–64	>32	10.0(6)	90.0(54)	2	16
Ciprofloxacin	0.12–64	>2	**73.3(44)** ^*c*^	26.7(16)	2	16
Moxifloxacin	0.12–8	>2	11.7(7)	88.3(53)	0.125	2
Rifabutin	0.12–8	>2	0.0(0)	100.0(60)	0.25	0.5
Linezolid	1–64	>16	8.3(5)	91.7(55)	2	8
Streptomycin	0.5–64	-^*a*^			8	32
Ethambutol	0.5–16	>4	**76.7(46)** ^*c*^	23.3(14)	8	16
Isoniazid	0.25–8	-^*a*^			2	8
SXT^*b*^	0.12–8	>2/38	31.7(19)	68.3(41)	0.5	8
Ethionamide	0.3–20	-^*a*^			0.6	2.5
Doxycycline	0.12–16	-^*a*^			16	16

a Only the MIC value reported, with no interpretation, for these drugs.

b SXT: trimethoprim-sulfamethoxazole.

c In boldface: resistance rate of ciprofloxacin and ethambutol to *M. kansasii* isolates was more than 70%.

### Mutation profiling.

The results of mutations are summarized in supplemental file 3. For EMB-resistant M. kansasii isolates and EMB-susceptible M. kansasii isolates, the sequences at the EMB resistance associated loci (*ebmB* and *embCA*) were identical. There were 304 amino acids different from those of M. tuberculosis H37Rv. All M. kansasii isolates had M306I and G406R substitution in the EMB resistance determining region, as it is referred to in M. tuberculosis. Compared with M. tuberculosis H37Rv, 12 mutations of *embCA* were observed in all M. kansasii isolates. All of these 60 M. kansasii isolates shared identical sequences of *rpoB*, *inhA*, *katG*, *rrl*, *rrs*, *rpsL*, *gyrA*, and *gyrB*.

## DISCUSSION

M. kansasii is the second most common cause of NTM disease in some regions of the United States, England, Wales, and France (17). In China, M. kansasii has been isolated from pulmonary infections in many areas, but the incidence is highest in the highly urbanized eastern and southern coastal regions (18, 19). From 2008 to 2012 in Shanghai, it was responsible for nearly half of all NTM infections (20).

M. kansasii subtype I was responsible for most human infections in Europe, the United States, and Japan (21, 22). Zhang et al. have shown that all of these methods, RFLP analysis with the major polymorphic tandem repeat probe and the IS1652 probe, PFGE, amplified fragment length polymorphism analysis, and PCR restriction analysis (PRA) of the *hsp65* gene, showed excellent typeability and reproducibility (21). In this study, the results of *hsp65* gene analysis were highly similar to those obtained with the *tuf* gene. All genotype I strains were isolated from patients with M. kansasii infections who met the ATS case definition criteria. Interestingly, all M. kansasii isolates were classified as molecular type I irrespective of the disease manifestation. In Brazil, Edson Machado et al. showed that 12 clinical M. kansasii isolates from Brazilian patients with pulmonary disease belonged to genotype I, as determined by *hsp65* sequencing (16). M. kansasii genotype I, as defined by PCR restriction-enzyme pattern analysis, is the most common genotype associated with human disease in China. In the present study, 98.3% of M. kansasii isolates were highly homogenous. In other Mycobacterium species, such small differences would be suggestive of the patients being epidemiologically linked. It has been reported that the municipal water supply is the main source of M. kansasii infections (23). We speculated that M. kansasii in this study may also originate from bacteria in other places that spread through water sources and were derived from the same ancestor as previously reported. However, it is unclear how waterborne M. kansasii could have achieved dissemination.

A previous study reported that M. kansasii subtype I contains five ESX systems, and the overall arrangement of the five ESX systems is similar to that in M. tuberculosis H37Rv (24). ESX-3, which encodes the proteins EsxG and EsxH, is required for optimal growth of M. tuberculosis and has been associated with essential processes such as iron and zinc acquisition. The *esxG* gene was absent in 56 M. kansasii isolates in the present study.

In addition, a previous study reported that *esxG* and *esxH* were upregulated in a drug-resistant M. tuberculosis isolate (25), an effect of EsxG and EsxH expression in mycobacterial RIF and INH resistance. However, *esxG* and *esxH* had no association with drug resistance of M. kansasii isolates in the present study.

The recommended therapy for M. kansasii infections is isoniazid (INH), RIF, and EMB. Treatment failure is almost always associated with RIF resistance. Previously described levels of resistance to RIF, established with the microdilution method, varied widely from 1.9% to 56.4% for RIF. In this study, resistance to RIF (20%) was found in 12 M. kansasii isolates. However, very high rates of RIF resistance were found in Iran (50.0%) and Beijing, China (56.4%) (18, 26). These differences could be related to the prevailing susceptibility pattern of M. kansasii isolates found in a particular geographical area. In addition, RIF has been shown to be unstable in current media used in phenotypic drug susceptibility testing of SGM (27, 28). RIF concentration in Middlebrook 7H9 medium had decreased by 92% after 7 days, and the microbiological assay revealed decreases in RIF concentration of ≥75% after 14 days (27). Resistance to RFB was not detected among the isolates in this study. RFB may be an alternative to RIF. In addition, INH may be useful clinically, but breakpoints for susceptibility and resistance for NTM have not been established. The proportion of isolates resistant to INH by microdilution method, using 1 mg/L INH as breakpoint, was 100% (29). The frequencies of INH resistance were 2.9% in Spain (30) and 8% in Brazil (31), with a breakpoint set at 5 mg/L, lower than those detected by this study, 26.7%. Consequently, susceptibility testing of INH in M. kansasii isolates may use 5 mg/L as the critical concentrations (32).

Notably, we found a very high rate of resistance to CIP (73.35%) and EMB (76.7%). This is similar to a report from da Silva Telles et al., who observed a high ciprofloxacin resistance rate (66%) and a high EMB resistance rate (94%) in Brazil (31). Previously described levels of resistance to CIP and EMB, established with the microdilution method, varied widely from 15% to 66% for CIP (26, 29, 31–33) and 0% to 100% for EMB (18, 26, 29–33). The high drug resistance rate may be associated with the widespread use of antitubercular drugs.

More recently, clarithromycin showed excellent activity against these microorganisms (98.33%), similar to previous studies that showed high *in vitro* activity of CLR against M. kansasii, with less than 1% of isolates showing resistance. High resistance (20.5%) of the M. kansasii isolates to CLR has been reported only in Beijing, China (18).

Additionally, AMK, LNZ, S, and rimethoprim-sulfamethoxazole (SXT) have good activity against M. kansasii *in vitro*. Resistance to AMK has seldom been reported in M. kansasii, with 0–5.1% of isolates resistant (18, 26, 31, 33), while high resistance was reported only in the Netherlands (54%) (29). The reason may be application of a lower breakpoint (5 mg/L). In previous studies, resistance to LNZ was not detected among M. kansasii isolates. Only in one study from Beijing, China, 25 (32.1%) of the isolates were resistant to LNZ, MIC_50_, and MIC_90_ values of 4 and 128 mg/L, respectively (18). However, our results (MIC_50_ = 2 mg/L and MIC_90_ = 8 mg/L) showed good activity, with 91.7% of isolates susceptible. LNZ may represent a good therapeutic alternative in M. kansasii infections, but there have been significant toxicity issues with this drug. The newer oxazolidinones have fewer side effects than LNZ and also have good *in vitro* activity against M. kansasii. With a breakpoint of 10 mg/L, resistance to S was 23.3% in this study, 14% in Brazil (31), and 40% in Greece (34). The MIC values of S (MIC_50_ = 8 mg/L, MIC_90_ = 32 mg/L) were similar to that reported from Poland (MIC_50_ = 8 mg/L) (32), while different from that of the U.S. (MIC_50_ = 2 mg/L) (35). SXT resistance detected in this study (31.7%) was higher than that reported in Poland (0%) (32) and Taiwan (18.9%) (33) using the same criteria. The reason may be its widespread use for controlling other microorganisms.

This research has several limitations. First, there were a limited number of M. kansasii isolates covered in this study. Second, further studies are required to conduct analysis of *esxG* and *esxH* genes and their secretion system.

In conclusion, these are preliminary data on the variability within the M. kansasii genotype I isolates in China. We evidenced four clusters that are separated by the presence of large numbers of SNPs throughout the genome. We found high activity of RFB, CLR, RIF, AMK, MXF, and LNZ against M. kansasii isolates. The high resistance rates observed with CIP and EMB should be cause for concern. Drug resistance in M. kansasii may have different genetic determinants than resistance to the same drugs in M. tuberculosis.

## MATERIALS AND METHODS

### M. kansasii isolates and genotyping.

Sixty M. kansasii isolates isolated from 60 patients were included in this study. All M. kansasii isolates were collected from 2018 to 2020 at a tertiary hospital, Shanghai Pulmonary Hospital in Shanghai, eastern China. Each isolate was obtained from the sputum or bronchial lavage of patients with M. kansasii pulmonary infection, based on the ATS guidelines for the diagnosis of M. kansasii infection (11). The epidemiological and clinical characteristics were obtained from medical records.

M. kansasii isolates were grown in Middlebrook 7H9 broth with OADC (0.85% sodium chloride, 5% bovine albumin, 2% dextrose, 0.003% catalase) at 37°C. Traditional species identification with para nitro benzoic acid and thiophene-2-carboxylic acid hydrazide (TCH) was performed to distinguish NTM from Mycobacterium tuberculosis complex. In parallel, all isolates were identified as M. kansasii by whole genome sequencing (WGS). PRA for the *hsp65* and *tuf* genes was carried out as previously described (36).

### Ethics statement.

The research protocol was approved by the Ethics Committee of Shanghai Pulmonary Hospital. We confirm that all adult subjects provided informed consent. Written or oral informed consent was obtained.

### Genome sequencing and assembly.

For WGS, genomic DNA from all 60 M. kansasii strains was extracted using the cetyl-trimethyl-ammonium bromide method, as described elsewhere (37). Extracted genomic DNA was quantified by Qubit 2.0 Fluorometer (Invitrogen, Carlsbad, CA, USA). Genomic DNA libraries were constructed using the Illumina TruSeq DNA Nano Library Prep Kit following the manufacturer’s protocol. The libraries were sequenced on a HiSeq X or a NovaSeq instrument (Illumina, San Diego, CA, USA) at a read length of 2 × 150 bp. The genomes were assembled into contigs using SPAdes (https://github.com/ablab/spades) (38), and the complete assemblies were annotated by Prokka (https://github.com/tseemann/prokka) (39).

### Genotyping and comparative genomics of M. kansasii.

In addition to the genomes of 60 M. kansasii strains sequenced in this study, genomes of the other 36 M. kansasii genome sequences available on the NCBI genome database (http://www.ncbi.nlm.nih.gov/genbank/) were downloaded for analysis to gain a better understanding of the genome epidemiology of M. kansasii. We aligned 96 genome sequences (60 from our study, 12 from Brazil, 4 from the U.S., 4 from Korea, 3 from Poland, 3 from the Netherlands, 2 from Japan, 3 from Switzerland, 2 from the Czech Republic, 2 from Germany, and 1 from the American Type Culture Collection, ATCC12478). The genomic sequences of these strains’ accession numbers are provided in supplemental file 2. For single-gene phylogenies, the respective sequences of each genetic locus were subjected to multiple alignment in MEGA X software (ClustalW algorithm). The resulting fragments were further trimmed to remove unnecessary gaps or regions using Trimal. These sequences were then used for evolutionary distance calculation according to the Jukes-Cantor model. Phylogenetic trees for each target locus sequence were built using the neighbor-joining method and midpoint rooted with the MEGA X software. Tree topologies were evaluated by bootstrap analysis based on 1,000 replications. The average nucleotide identity (ANI) was calculated by OrthoANI (40). The pairwise ANI values were determined from using pyani (https://github.com/widdowquinn/pyani) and visualized using the complex heatmap R package (41). Single nucleotide polymorphisms (SNPs) were identified using snippy (https://github.com/tseemann/snippy), and a pseudo-genome alignment was generated. Recombined regions were detected using Gubbins. A phylogenetic tree was constructed using the SNPs outside of the recombination regions with RAxML using a GTR model and gamma correction. The output from Gubbins was used as the input for BactDating v1.0 to perform phylogenetic dating based on a Bayesian approach. The Markov chain Monte Carlo chain lengths were run for 100 million cycles to convergence; the effective sample size of the inferred parameters α, μ, and σ was >200. Antimicrobial resistance genes were mined using AMRFinderPlus v3.9.8. Virulence genes were identified by ABRicate v1.01 (https://github.com/tseemann/abricate) using the VFDB database (http://www.mgc.ac.cn/VFs/main.htm) with 85% identity and 85% query coverage cutoffs.

### Antimycobacterial drug susceptibility testing.

Antimicrobial susceptibility testing was performed following the guidelines of the CLSI (13). A panel of 13 antimicrobials using Sensititre SLOMYCO plates (Thermo Fisher Scientific, USA) were assessed according to the manufacturer’s instructions. The antimicrobial agents and the concentrations tested were as follows: amikacin (AMK), 1–64 μg/mL; ciprofloxacin (CIP), 0.12–16 μg/m; clarithromycin (CLA), 0.06–64 μg/mL; moxifloxacin (MXF), 0.12-8 μg/mL; trimethoprim-sulfamethoxazole (SXT), 0.12–8 μg/mL; linezolid (LZD), 1–64 μg/mL; doxycline (DOX), 0.12–16 μg/mL; streptomycin (S), 0.5–64 μg/mL; rifampin (RIF), 0.25–8 μg/mL; rifabutin (RFB), 0.12–8 μg/mL; isoniazid (INH), 0.25–8 μg/mL; ethambutol (EMB), 0.5–16 μg/mL; and ethionamide (ETH), 0.3–20 μg/mL (Table 1). M. kansasii strain ATCC 12478 was used as a reference strain for MIC testing. MIC_50_ and MIC_90_ values were derived from MIC distribution.

### Statistical analysis.

All statistical analyses were performed using SPSS software, version 24 (IBM, USA). The MIC distributions were analyzed using GraphPad Prism software (version 7.00, La Jolla, CA, USA).

### Data availability.

The Illumina sequences of the 60 M. kansasii isolates in this study are available in the Sequence Read Archive (BioProject ID: PRJNA780966). The accession numbers are listed in supplemental file 1.
