# Supplementary material for: Clinical and Microbiological Characteristics of Mycobacterium kansasii Pulmonary Infections in China
Source: Microbiol Spectr. 2022 Jan 12;10(1):e01475-21. doi: 10.1128/spectrum.01475-21 (PMC8754148; doi:10.1128/spectrum.01475-21)
Supplement: SUPPLEMENTAL FILE 1 — Supplemental material. Download SPECTRUM01475-21_Supp_1_seq10.pdf, PDF file, 0.3 MB [file spectrum01475-21_supp_1_seq10.pdf]

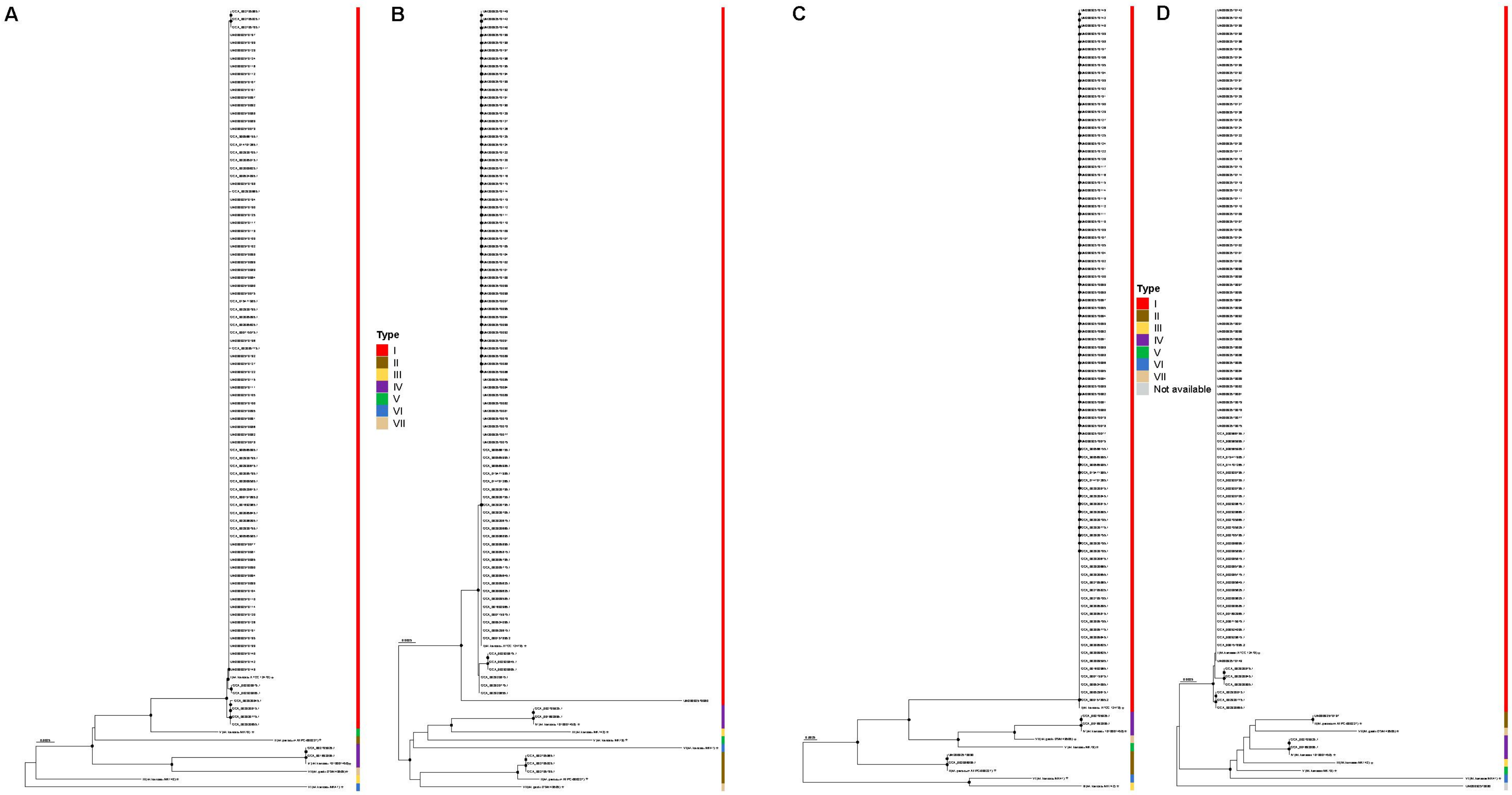

A and B: All 60 isolates tested were classified as *M. kansasii* type I based on *rpoB* and *hsp65* gene sequences. C and D: Fifty-nine isolates exhibited 98.3% identity at both loci when compared with the *M. kansasii* genotype I reference strain (ATCC12478) based on ITS and *tuf* gene sequences.
